# Magnetic Resonance Imaging to Evaluate the Recovery Effects of Cerebral Nerve Function in Comprehensive Treatment of Poststroke Depression by Intelligent Algorithm-Based Hyperbaric Oxygen Therapy

**DOI:** 10.1155/2022/6214223

**Published:** 2022-03-30

**Authors:** Chunhua Yuan, Lan Zhang, Yankun Hao, Jun Liang, Tingting Ma

**Affiliations:** ^1^Department of Neurology, The Second Affiliated Hospital of Mudanjiang Medical University, Mudanjiang 157000, China; ^2^Student Affairs Department, Mudanjiang Medical University, Mudanjiang 157011, China; ^3^Department of Medical Function, Mudanjiang Medical University, Mudanjiang 157011, China; ^4^Stem Cell Institute, Mudanjiang Medical University, Mudanjiang 157011, China; ^5^Department of Clinical Psychology, Mudanjiang Medical University, Mudanjiang 157011, China

## Abstract

This research was aimed to discuss magnetic resonance imaging (MRI) evaluation of recovery effects of cerebral nerve function in comprehensive treatment of poststroke depression (PSD) by intelligence-based hyperbaric oxygen therapy. Low-rank matrix algorithm was adopted to denoise MRI images of patients with PSD, and mean square error (MSE) and peak signal-to-noise ratio (PSNR) were the evaluation indicators of the results of image denoising. 118 patients were randomly divided into the control group (administered escitalopram oxalate) and the research group (hyperbaric oxygen therapy was implemented based on the treatment in control group). National Institutes of Health Stroke Scale (NIHSS), Hamilton Depression Scale (HAMD), Pittsburgh Sleep Quality Index (PSQI), glial fibrillary acidic protein (GFAP), and changes of norepinephrine (NE) level of patients in two groups were compared before and after treatment. The value of MSE of MRI images processed by low-rank matrix algorithm was 92.39, which was higher than that calculated by nonlocal mean (NLM) algorithm (80.54). The PSNR value calculated by low-rank matrix algorithm was 25.35, which was lower than that calculated by NLM algorithm (29.07). In contrast, NIHSS score and HAMD score of the research group after treatment were lower than those of the control group, while PSQI score of the research group was higher than that of the control group. The level of GFAP of the research group was at 852.46 ± 94.47, which was significantly lower than that of the control group, reaching 948.53 ± 98.42. However, the level of NE of the research group was 1478.59 ± 99.85, which was higher than that of the control group (1061.80 ± 98.02). All the comparisons of above indicators had statistical meaning (*P* < 0.05). The low-rank matrix algorithm can help in clinical diagnosis and treatment to provide more accurate MRI images. In addition, hyperbaric oxygen comprehensive therapy can promote the recovery of neurological function in patients with poststroke depression and significantly improve the depressive state and sleep quality of patients.

## 1. Introduction

Cerebral stroke is the third main cause of death following coronary heart disease and cancer throughout the world, and its incidence is growing year by year [1]. Poststroke depression (PSD) refers to a kind of mental disease featured with low mood and loss of interest after cerebral stroke occurs, and it is the mental complication with the highest incidence among all cerebral poststroke complications. PSD has greatly adverse effects on the comprehensive recovery of cerebral stroke patients, including the extension of hospitalization, the rise of mortality, and the influence of physical and verbal rehabilitation [2]. Many clinical reports show that the incidence of PSD is high, ranging from 25% to 65% generally [3]. Cerebral PSD with high incidence may occur during the acute phase of cerebral stroke and 1 to 2 years after stroke. According to domestic medical experts, the incidence is generally about 34% [4]. Some other studies demonstrate that depression may lead to vascular dementia [5]. Drug treatment is the main therapeutic method in medication treatment of PSD, but the adverse reactions to drug is very obvious. In addition, nondrug treatments such as transcranial magnetic stimulation therapy, electroconvulsive therapy, and exercise therapy have problems such as slow onset, many side effects, and high price, which discourage many patients [6].

Related research demonstrated that hyperbaric oxygen (HBO) treatment could promote the diffusion of oxygen into brain tissue to protect nerve by increasing the partial pressure of oxygen [7]. Vataja et al. [8] showed in related studies that the lesions in different brain areas were closely related to the incidence of patient's emotions (depression and anxiety disorder) after cerebrovascular accident. Alexopoulos et al. [9] pointed out in related studies that diabetes and coronary heart disease atrial fibrillation were related to the depression after cerebrovascular accident. Besides, related studies showed that the signal of brain white matter abnormality was associated with depression, whose source could be traced in terms of blood vessels [10, 11]. Nuclear magnetic resonance imaging (MRI) could observe the morphological changes of PSD by displaying the features of stroke lesions and acquire the information about neurophysiology, biochemistry, structure, and function of PSD from different levels [12]. MRI denoising algorithm aimed at reducing noise power, but RI itself contained special information, and these details needed to be retained as much as possible in processing these images [13]. Some scholars found out that low-rank matrix algorithm could eliminate the noises in low-dosage computerized tomography (CT) images effectively, which indicated that low-rank matrix algorithm was worthy of application [14]. At present, there are few data on the adoption of this algorithm in MRI of cerebral stroke. Based on the current situation, the research aimed at providing some references for more comprehensive retrieval of MRI information about cerebral stroke patients in clinical diagnosis and treatment for doctors by researching the denoising of this algorithm in MRI of cerebral stroke patients.

## 2. Data and Methods

### 2.1. Research Objects

In this research, 118 patients with cerebral stroke in the hospital from June 2017 to September 2020 were selected as the research objects. Among these patients, 65 cases were males and 53 cases were females aged 50–77 years old. All patients were randomly divided into control group and research group, with 59 cases in each group. Escitalopram oxalate was offered to treat patients in the control group, and patients in the research group received HBO treatment based on the same treatment with the control group. This research had been approved by ethics committee of the hospital. All patients as well as their family members were informed, and they signed the informed consent forms.

Inclusion criteria were as follows. (a) All patients who met the diagnosis standards set in the 4th National Cerebrovascular Academic Conference in 1995 and the diagnosis standards related to depression of capability maturity model integration-level 3 (CCMI-3). (b) All patients diagnosed with cerebral hemorrhage or cerebral infarction by CT examination. (c) Patients without contraindication to MRI. (d) Patient whose MRI data were complete and images were clear and distinguishable.

Exclusion criteria were as follows. (a) Patients with unstable vital signs. (b) Patients with aphasia and inability to engage in the evaluation of neurological deficit and psychological assessment of depression. (c) Patients who could not cooperate in MRI examination due to mental diseases. (d) Intolerance to HBO or contraindications. (e) Patients with other previous mental diseases.

### 2.2. MRI Examination

The MRI imaging system and 8-NV combined head and neck coil were adopted. Patients were asked to lie on their backs and to fix their heads and necks within the coil. Next, high-pressure injectors were connected. The neck vascular system was scanned by 3D time of flight (3DTOF), and then carotid stenoses were observed by multi-information platform (MIP) and multi-planar reformation (MPR) image reconstruction. Coronal (COR) bit three-dimensional T1-weighted image (3D-T1WI) in lumen stenoses was scanned horizontally, time-resolved spectrum (TRS) bit platelet distribution width (PDW) phase was scanned, and TRS bit T2-weighted image (T2WI) was scanned. After that, contrast-enhanced magnetic resonance angiography was conducted followed by mask scanning. Next, contrast agent Gadopentetic acid (GA) was injected. Once three blood vessels were shown above aortic arch under fluoroscopy, 3D scan should be started right away. Finally, enhancement scanning was performed by 3D-T1WI. The scan parameters were set as follows. Firstly, the sites where cerebral infarction newly occurred included the frontal lobe, temporal lobe, parietal lobe, occipital lobe, radiation crown area, semioval center, thalamus, basal ganglia and inner capsule, and brain stem and cerebellum. The scanning parameters were set as follows: (i) the new cerebral infarction sites were divided into frontal lobe, temporal lobe, parietal lobe, occipital lobe, corona radiata, centrum semiovale, thalamus, basal ganglia and internal capsule, and brain stem and cerebellum; (ii) the number and volume of new cerebral infarction; (iii) the number of old cerebral infarction; (iv) brain atrophy was assessed by ventricle-to-brain ratio (VBR); (v) white matter lesions (WMLs) were measured by fluid attenuated inversion recovery (FLAIR) sequence, and deep white matter hyperintensities (DWMH) and periventricular hyperintensities (PVH) were scored by Fazekas.

### 2.3. Denoising of Images Based on Low-Rank Matrix Algorithm

The rank of matrix was defined mathematically as the ordinal number of the largest sub-determinant whose determinant was not zero in a matrix and the maximum number of rows or columns irrelevant to linearity in rows or columns of a matrix. Therefore, the rank could reflect the correlation between rows or columns in a matrix comprehensibly. In recent years, the denoising of images by low-rank matrix has become a hot topic. Low rank was guaranteed by kernel norm constraint, and the sparsity was kept to restore images by constraining the sparsity of noises, which aimed at denoising. The model of the denoising by low-rank matrix was presented by mathematical definition, which was shown in the following equation:

(1) $$\begin{array}{c} S=R+H, \end{array}$$

where *S* referred to noisy images, *R* represented true and noiseless images with low rank, and *H* meant noise matrix. If *H* stood for Gaussian white noises, low-rank matrix *R* could be solved by principal component analysis (PCA). If *S*, *R*, *H* ∈ *N*^*m*×*n*^,  *a*_*i*,*j*_ ∈ *H*(0 ≤ *i* ≤ *m*, 0 ≤ *j* ≤ *n*), the true image *R* was derived by the model shown by

(2) $$\begin{array}{c} \underset{I,H}{\min}{\left\|H\right\|}_{F}\\ \text{s.t.}\text{ trank}\left(R\right)\leq \text{r},\;S=R+H, \end{array}$$

where ${\left\|H\right\|}_{F}=\sqrt{\left(\sum_{i=1}^{n}\sum_{j=1}^{n}\left|a_{i,j}\right|\right)}$ referred to the Frobenius norm of the matrix *H* and *r* << min(*m*, *n*) meant the rank of the matrix *R*. When the noise level of noise image *S* was low, the vectors corresponding to the first *r* largest singular values were obtained and then the optimal solution of the image *R* was obtained by the processing of *S* by PCA. When the noise level of noise image *S* was high and sparse, the denoising results of the denoising model shown in (2) contained lots of residual noises, and insufficient denoising occurred. However, insufficient denoising could be avoided by using robust PCA algorithm, whose specific algorithm model was expressed by

(3) $$\begin{array}{c} \underset{I,H}{\min}\left[{\left\|H\right\|}_{0},s.t.\text{rank}\left(R\right)\right],\\ \text{s.t. }S=R+H. \end{array}$$

Because *R* and *H* were both unknown variates and the focus of the solution of different questions in actual process differed in different cases, the hyperparameter *α* was introduced into the model to balance the sparsity of noise matrix *H* and the low rank of the true image *R.* (3) was transformed into (4) as follows:

(4) $$\begin{array}{c} \underset{I,H}{\min}\alpha {\left\|H\right\|}_{0}+\text{rank}\left(R\right)\\ \text{s.t. }S=R+H. \end{array}$$

The norm *y*_*0*_ of the matrix needed to be solved in (4). Convex relaxation was performed on (4), the norm *y*_*0*_ was replaced by the norm *y*_*1*_ in the matrix to figure out approximate sparse solution, and then the rank (*x*) was replaced by kernel norm to solve low rank. At the end, equation (5) was generated as follows.

(5) $$\begin{array}{c} \underset{I,H}{\min}\alpha {\left\|H\right\|}_{1}+{\left\|R\right\|}_{*}\\ \text{s.t. }S=R+H, \end{array}$$

where ||*R*||_*∗*_ meant the nuclear norm, which was the sum of all singular values in matrix *R*. The definition of this nuclear norm is shown in the following equation:

(6) $$\begin{array}{c} {\left\|R\right\|}_{*}=\text{trace }\left(\sqrt{R^{T}R}\right)=\sum_{i=1}^{\min\left(m,n\right)}\beta_{i}, \end{array}$$

where trace (*x*) referred to the trace operator that summed singular values of matrix and *β*_*i*_ was the *i*th singular value of *R*.

### 2.4. HBO Treatment Method

Escitalopram oxalate was offered in the treatment of patients in the control group. The initial dosage was 10 mg/d. Based on the observation of patients' tolerance of one week of drug use, the dosage was increased to 20 mg/d and patients were asked to take for consecutive 7 weeks if no adverse reactions occurred.

Based on the same treatment with patients in the control group, HBO treatment was offered to patients in the research group. Multi-person air pressurized cabin was adopted in the treatment. Pressurization and decompression at constant speed lasted both for 20 minutes. The pressurization value in the treatment was 0.15 MPa. Patients needed to breathe oxygen wearing masks for 60 minutes with an interval of 10 minutes. Oxygen inhalation occurred one time every day, and 10 times of oxygen inhalation constituted one course of treatment. According to individual diseases, 2 to 4 treatment courses were appropriate for different patients. The curative effects on patients in two groups were assessed 7 weeks after the treatment.

### 2.5. Evaluation of Results of Denoising Algorithm

To assess the performance of the algorithm quantitatively, mean square error (MSE) and peak signal-to-noise ratio (PSNR) were selected as the technical indicators of the evaluation of the results of image denoising. MSE describes the differences between ideal reference images and images to be evaluated. A small MSE value means that the images to be assessed are similar to ideal images with high quality. The differences are calculated by (7). PSNR is defined by MSE. A large value of PSNR indicates that the images to be assessed are similar to ideal images with high quality. PSNR values are calculated by (8).

(7) $$\begin{array}{c} \text{MSE}\left(a,b\right)=\frac{1}{AB}\sum_{i=1}^{A}\overset{B}{\underset{j=1}{\sum }}{\left[f\left(i,j\right)-g\left(i,j\right)\right]}^{2}, \end{array}$$

(8) $$\begin{array}{c} \text{PSNR}\left(a,b\right)=10\;\log_{10}\left(\frac{L^{2}}{\text{MSE}\left(a,b\right)}\right), \end{array}$$

where *a* refers to ideal reference images and *b* represents images to be assessed. The size of these images is *A* × *B*, and L means peak signal.

### 2.6. Surgical Observation Indicators

The following assessments were made based on the observation on pretherapeutic and posttherapeutic indicators of patients in two groups. *(a) Nerve Function Assessment*. The nerve function of patients was evaluated by National Institutes of Health Stroke Scale (NIHSS) of prevention and treatment guide on neurological diseases in China. The nerve function was scored based on level of consciousness, instruction compliance, eye movement, and verbal expression. The full score was 50. The higher the score, the more sever the nerve defect. Among different score levels, it meant normal nerve function when the score was less than 1, it meant slight stroke when the score was 2∼10 points, it meant moderate stroke when it was 11∼25 points, it demonstrated moderate-severe stroke when it was 26∼35, and it meant severe stroke when it was 36∼50. *(b) Assessment of Degree of Depression*. Based on Hamilton Depression Scale (HAMD), total score higher than 24 represented severe depression, scores between 17 and 24 meant slight-moderate depression, and scores lower than 17 indicated no depression. *(c) Assessment of Sleep Quality*. The sleep quality of all patients was scored by Pittsburgh Sleep Quality Index (PSQI). The total score ranged from 0 to 20. High score represented high sleep quality. *(d) Changes of Level Indicators of Glial Fibrillary Acidic Protein (GFAP) and Norepinephrine (NE)*. The levels of GFAP and NE in blood samples of patients in two groups were measured by enzyme-linked immunosorbent assay (ELISA).

### 2.7. Experimental Process

Plasma GFAP levels were determined by ELISA. Peripheral blood was collected from stroke patients immediately after admission, and 4.8 mL of venous blood was collected from the normal control group during physical examination. The blood samples were placed in anticoagulant tubes, centrifuged for 3000 min at room temperature for 16 min, and the plasma was collected and stored at −82°C for examination. The plasma GFAP concentration was determined by ELISA, and the absorbance value of the samples was determined by the Bio-Rad 680 absorbance method. The standard curve and concentration of plasma collagen fibrillary acidic protein were calculated according to the kit instructions, expressed in ng/L.

### 2.8. Statistical Methods

Data were analyzed statistically by Statistical Product and Service Solutions (SPSS) 20.0 software. Measurement data were expressed by mean value ± standard deviation (‾*x* ± *s*), *t* was adopted in testing, enumeration data were tested by chi-square test, and *P* < 0.05 indicated that the differences were significant and had statistical meaning.

## 3. Results

### 3.1. General Data

The indicators of gender, age, basic medical history (hypertension and diabetes), and previous stroke history of 118 patients included in the research showed no statistical meaning (*P* > 0.05) (Table 1).

### 3.2. Results of Image Denoising

**Figure 1:**
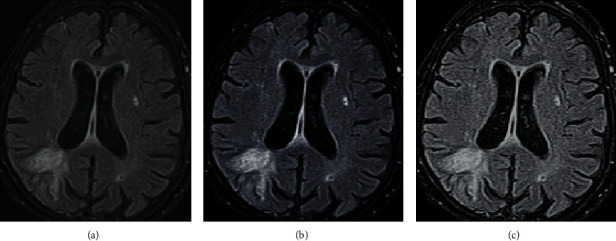
Results of denoising of MRI images of cerebral stroke patients by each algorithm. (a) The original image. (b) The results of denoising by NLM algorithm. (c) The results of denoising by low-rank matrix algorithm.

Figure 1 shows the denoising results.  Figure 1(a) shows the original image, Figure 1(b) demonstrates the results of denoising by NLM algorithm, and Figure 1(c) presents the results of denoising by low-rank matrix algorithm in the research. According to Figure 1(c), the results of denoising by low-rank matrix algorithm in the research were ideal because of clear outline and high distinguishability.

### 3.3. Evaluation of Results of Denoising

**Figure 2:**
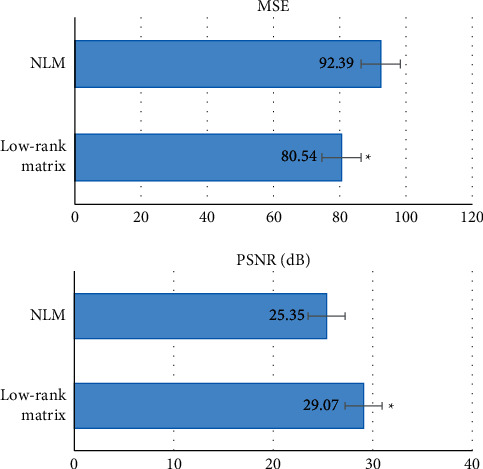
Comparison of evaluation indicators of denoising of MRI images by each algorithm. ^∗^ indicated a statistically significant difference between groups (*P* < 0.05).

To compare the denoising performance of low-rank matrix in the research in MRI images quantitatively, MSE and PSNR were adopted to assess the performance of algorithm in images. The results of the assessment are shown in Figure 2. The MSE value of processing MRI images by low-rank matrix algorithm in the research was 92.39, which was higher than that presented by NLM algorithm (80.54). Besides, the PSNR value presented by low-rank matrix algorithm was 25.35, which was lower than that presented by NLM algorithm (29.07). The results demonstrated that the denoising effects on MRI images by low-rank matrix algorithm in the research were better compared with NLM algorithm.

### 3.4. Assessment Results of Nerve Function

**Figure 3:**
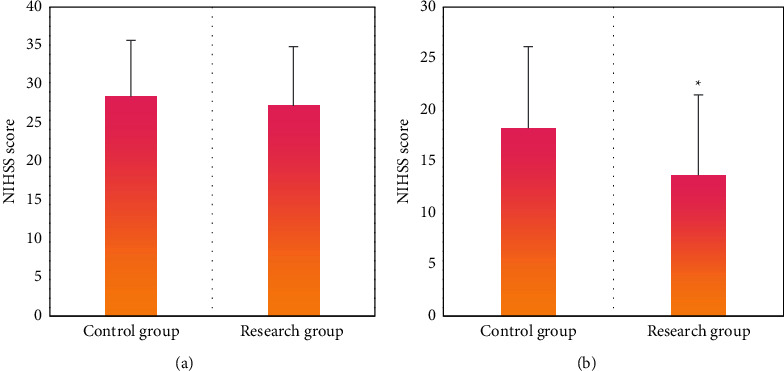
Scoring results of NIHSS of patients in two groups before and after treatment. (a) The scoring results before treatment. (b) The scoring results after treatment. ^∗^indicated a statistically significant difference between groups (*P* < 0.05).

Figure 3 demonstrates the scoring results of NIHSS of patients in two groups before and after treatment. According to Figure 3, the NIHSS scores of patients in two groups before treatment showed no obvious differences. In contrast, NIHSS score of the research group after treatment was 13.68 ± 7.81, which was significantly lower than that of the control group (18.25 ± 7.92) (*P* < 0.05). The differences indicated that the nerve function of patients in the research group was better than patients in the control group after treatment.

### 3.5. Results of Assessment of Degree of Depression

**Figure 4:**
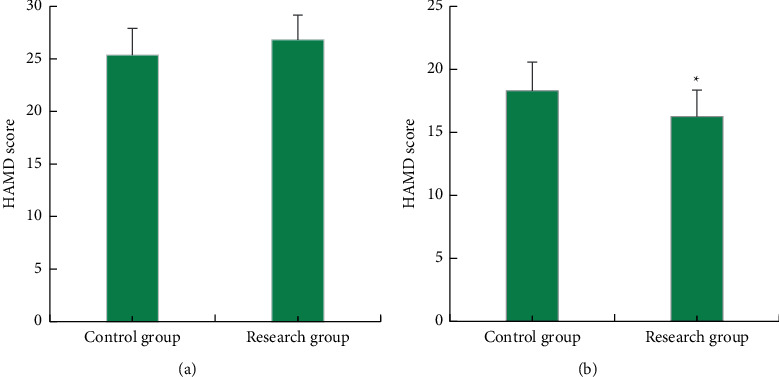
Results of HAMD scores of patients in two groups before and after treatment. (a) The scoring results before treatment. (b) The scoring results after treatment. ^∗^indicated a statistically significant difference between groups (*P* < 0.05).

Figure 4 illustrates scoring results of HAMD of patients in two groups before and after treatment. According to Figure 4, the HAMD scores of patients in two groups before treatment demonstrated no significant differences. In contrast, HAMD score of the research group after treatment was 16.25 ± 2.10, which was obviously lower than that of the control group (18.30 ± 2.27) (*P* < 0.05). The differences showed that the depression of patients in the research group was improved significantly than that of patients in the control group.

### 3.6. Results of Assessment of Sleep Quality

**Figure 5:**
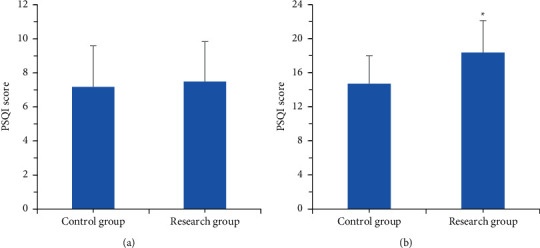
Results of PSQI scores of patients in two groups before and after treatment. (a) The scoring results before treatment. (b) The scoring results after treatment. ^∗^indicated a statistically significant difference between groups (*P* < 0.05).

Figure 5 shows the scoring results of PSQI of patients in two groups before and after treatment. According to Figure 5, the comparison of PSQI scores of patients in two groups before treatment showed no significant differences. In contrast, PSQI score of the research group after treatment was 18.36 ± 3.75, which was obviously higher than that of the control group (14.70 ± 3.28) (*P* < 0.05). The differences showed that the sleep quality of patients in the research group was higher than that of patients in the control group.

### 3.7. Results of Comparison of Levels of GFAP and NE in Serum between Patients in Two Groups

**Figure 6:**
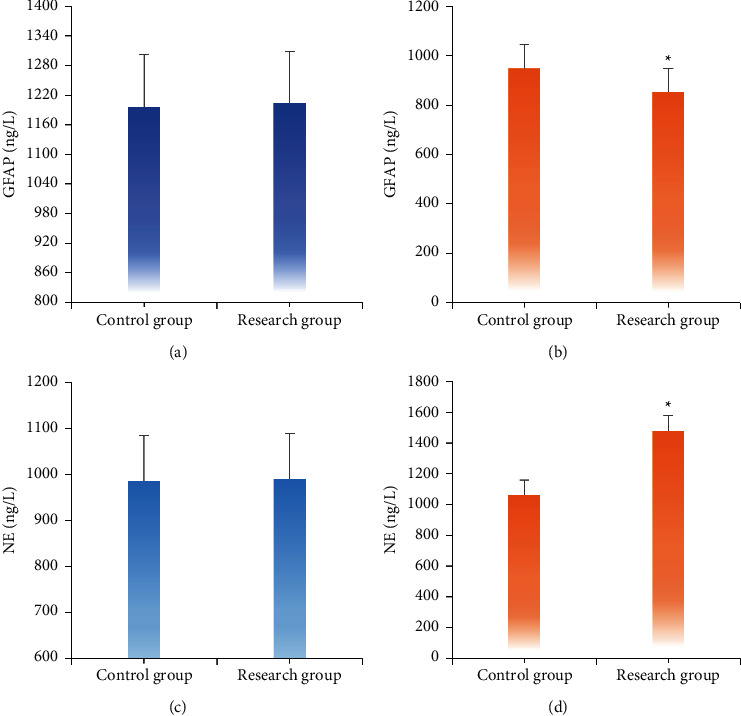
Comparison of changes of GFAP and NE levels in serum of patients in two groups before and after treatment. (a, c) The scoring results before treatment. (b, d) The scoring results after treatment. ^∗^ indicated a statistically significant difference between groups (*P* < 0.05).

Figure 6 demonstrates the comparison of GFAP and NE levels of patients in two groups before and after treatment. According to Figure 6, the GFAP and NE levels of patients in two groups before treatment showed no significant differences. In contrast, GFAP level of the research group after treatment was 852.46 ± 94.47, which was obviously lower than that of the control group (948.53 ± 98.42) (*P* < 0.05). Besides, NE level of patients in the research group was 1478.59 ± 99.85, which was higher than that of the control group (1061.80 ± 98.02).

## 4. Discussion

PSD is a common complication of cerebral stroke [15], which causes the increase of disability rate and fatality rate. As a result, patients' life quality decreases. It is a severe complication affecting prognosis [11, 16]. Related studies showed that the incidence of cerebral PSD was related to neurotransmitter deficiency. Cerebral stroke damaged NE cholinergic neuron and its path, so neurotransmitter became rare, which resulted in depression. Therefore, the depression during acute cerebral stroke phase was more likely to be determined by biological factors. Neural factors like GFAP and NE levels were firmly related to patients' depression [17]. The symptomatic treatment was the main therapeutic method of treating cerebral PSD, such as anti-depression drug treatment. Some gerontal patients had chronic diseases, including heart, lung, liver, and kidney diseases other than previous symptoms. The adverse reactions to drug among elderly patients were obvious, and they could hardly adapt to drug effects, which greatly affected clinical effects. Hyperbaric oxygen treatment in cerebral PSD was reported domestically recently. However, there was no more explanations for the treatment mechanism. Patients with cerebral PSD were treated by the HBO method in the research, and the results showed that NIHSS and HAMD scores of the research group were obviously lower than those of the control group after treatment, while PSQI score of the research group was higher than that of the control group. The differences demonstrated that HBO could promote the recovery of nerve function of patients and improve depression and sleep quality of patients significantly.

Some studies showed that the changes of levels of related cytokines in body played an important role in the incidence of PSD and cognitive impairment [18]. GFAP is a special protein which constituted astroglial cells. The main physiological function of GFAP is to maintain blood-brain barriers and to protect nerve system against the invasion of harmful substance [19]. Meanwhile, it also played an essential role in the transmission of neuron signals and cognitive function. NE is formed after N-methyl is removed from epinephrine. It played a vital role in sympathetic nervous excitement [20]. Previous studies demonstrated that nerve factors like GFAP and NE levels were closely related to patients' depression [21]. Besides, GFAP level of patients in the research group was lower than that of patients in the control group, but NE levels of patients in the research group was higher than that of patients in the control group. The differences indicated that HBO could improve patients' neurological deficit, promote the recovery of patients' nerve function, enhance nerve system excitability, and further alleviate patients' depression.

At present, there is no final conclusion in the pathogenesis of PSD. However, the development of magnetic resonance software and hardware technology enables MRI to present neurophysiology, biochemistry, structure, and function of PSD from different levels. Furthermore, MRI has unique function in studying the intrinsic pathological mechanism of PSD. Nevertheless, MRI scan lasts long, and artefacts usually appear in images, which often affects doctors' identification of lesions. With the rapid development of intelligent algorithms, this problem could be solved. Low-rank matrix algorithm could denoise and make images clear by removing artefacts from images. This algorithm was adopted to denoise MRI images of cerebral stroke patients in the research. The results demonstrated that the MSE value of processing MRI images by low-rank matrix algorithm was 92.39, which was higher than that presented by NLM algorithm (80.54). Besides, PSNR value presented by low-rank matrix algorithm was 25.35, which was lower than that presented by NLM algorithm (29.07). The differences indicated that low-rank matrix algorithm in the research had obvious effects on image denoising because the outlines of the denoised images were clear and images were distinguishable. It is concluded that the low-rank matrix algorithm can help in clinical diagnosis and treatment to provide more accurate MRI images. Hyperbaric oxygen comprehensive therapy can promote the recovery of neurological function in patients with poststroke depression and significantly improve the depressive state and sleep quality of patients.

## 5. Conclusion

Low-rank matrix algorithm was adopted to denoise MRI images of cerebral PSD patients in the research, and the obvious denoising effects on MRI images by this algorithm were confirmed because the denoised MRI images of cerebral PSD patients became clear and accurate MRI images were offered for clinical diagnosis and treatment. HBO comprehensive treatment could promote the recovery of nerve function of cerebral PSD patients and improve patients' depression and sleep quality. In addition, the improvement might be related to the decline of GFAP level in serum and the increase of NE level. The denoising effect of the algorithm in MRI images of stroke patients can provide doctors with greater access to MRI image information of stroke patients in clinical diagnosis and treatment. There were also many deficiencies in the research. The sample size was relatively small, and more experimental people should be included, not in a single area or in a small area. Clinical trials should be conducted in multi-center, large-sample hospitals.

## Figures and Tables

**Table 1:** Results of comparison of general data on all patients.

Group	Gender (men/women)	Age (years old)	Basic medical history		Previous stroke history (*n*)
			Hypertension (*n*)	Diabetes (*n*)	
Control group	31/28	63.26 ± 10.43	32	40	20
Research group	33/26	63.38 ± 12.52	43	39	24
*P* value	0.857	0.392	0.466	0.609	0.746

## Data Availability

The data used to support the findings of this study are available from the corresponding author upon request.
